# Preoperative controlling nutritional status (CONUT) score as a predictor of long-term outcome after curative resection followed by adjuvant chemotherapy in stage II-III gastric Cancer

**DOI:** 10.1186/s12885-018-4616-y

**Published:** 2018-06-28

**Authors:** Xuechao Liu, Deyao Zhang, Enzi Lin, Yongming Chen, Wei Li, Yingbo Chen, Xiaowei Sun, Zhiwei Zhou

**Affiliations:** 1 State Key Laboratory of Oncology in South China; Collaborative Innovation Center for Cancer Medicine, Sun Yat-sen University Cancer Center, Guangzhou, 510060 China; 2 Department of Gastric Surgery, Sun Yat-sen University Cancer Center, 651# East Dongfeng road Guangzhou, 510060 Guangdong Province, People’s Republic of China; 3 Cancer Hospital of Shantou University Medical College, Shantou, 515041 China

**Keywords:** CONUT score, PNI, Adjuvant chemotherapy, Gastric cancer, Prognosis

## Abstract

**Background:**

The prognostic value of preoperative controlling nutritional status (CONUT) has been reported in many malignancies. In present study, we aimed to clarify the prognostic impact of CONUT in gastric cancer (GC) receiving curative resection and adjuvant chemotherapy.

**Methods:**

We retrospectively reviewed 697 consecutive patients undergoing curative surgery followed by adjuvant chemotherapy for Stage II-III GC between November 2000 and September 2012. Patients were classified into high (≥3) and low (≤2) CONUT groups according to the receiver operating characteristic (ROC) analysis.

**Results:**

Of the included patients, 217 (31.1%) belonged to the high CONUT group. The high CONUT group had a significantly lower 5-year cancer-specific survival (CSS) rate than the low CONUT group (39.3 vs. 55.5%, *P* < 0.001). High CONUT score was significantly associated with larger tumor size, more lymph node metastasis, and poorer nutritional status, including lower body mass index (BMI), higher prognostic nutritional index (PNI) and the presence of preoperative anemia (all *P* < 0.05). Multivariate analysis revealed that CONUT score was an independent prognostic factor (HR: 1.553; 95% CI: 1.080–2.232; *P* = 0.017). Of note, in the low PNI group, CONUT score still effectively stratified CSS (*P* = 0.016). Furthermore, the prognostic significance of CONUT score was also maintained when stratified by TNM stage (all *P* < 0.05).

**Conclusions:**

CONUT score is considered a useful nutritional marker for predicting prognosis in stage II-III GC patients undergoing curative resection and adjuvant chemotherapy, and may help to facilitate the planning of preoperative nutritional interventions.

**Electronic supplementary material:**

The online version of this article (10.1186/s12885-018-4616-y) contains supplementary material, which is available to authorized users.

## Background

Gastric cancer (GC) is the third most common cause of cancer death and a major public health problem worldwide. In China, despite the decreasing incidence trend of GC, population growth and ageing still lead to a large and rising number of new cases in recent years [1, 2]. To better achieve the clinical outcome, surgical technique, chemotherapies and targeted therapy have improved [3]. Recently, there is increasing interest for clinicians to identify prognostic factors for tailored treatment.

One such factor that has arisen substantial attention is the nutritional and immunological status, which is reported to be associated with the clinical outcomes in various malignancies [4–6]. Several preoperative scoring systems are developed to assess nutritional risk, postoperative complications and long-term outcomes, such as the prognostic nutritional index (PNI), subjective global assessment, and Nutritional Risk Index [7–9]. The controlling nutritional status (CONUT) score, another screening tool for nutritional status, is calculated from the serum albumin concentration, total cholesterol level and total peripheral lymphocyte count, which are representative markers of protein reserves, calorie deficiency, and impaired immune defenses, respectively [10]. Serum albumin concentration is not only a major indicator of nutritional status but also an important determinant of the immune response. Hypoalbuminemia has been reported to be associated with poor outcome in various malignancies, including GC [11, 12]. Total cholesterol level also has been revealed to correlate with tumour progression and prognosis in many types of cancers [13]. In addition, lymphocytes play a key role in cell-mediated immunity and are thought to initiate a cytotoxic immune response by inducing cell apoptosis, suppressing tumor cell proliferation, invasion, and migration [14]. The combination of the three components into CONUT may better reflect the balance of nutritional status and enhance the ability to accurately predict general condition.

Recently, CONUT score has been demonstrated as a predictive or prognostic marker in many types of cancers [15–17]. Of note, a study from Japan showed that CONUT was useful for predicting long-term outcome in pStage I-II, but not in pStage III GC patients [18]. Due to the regional differences as well as different multidisciplinary treatment mode, the impact of CONUT score on prognosis in GC patients undergoing curative resection and adjuvant chemotherapy remains unclear.

In this study, we performed a sufficiently large, representative and consecutive sample to evaluate the prognostic value of the preoperative CONUT score, along with several common nutritional markers including PNI, body mass index (BMI), performance status and preoperative anemia.

## Material and methods

### Patients

We retrospectively reviewed the medical records of 697 consecutive patients undergoing open D2 radical gastrectomy with R0 resection at Sun Yat-sen University Cancer Center, from November 2000 to September 2012. All patients had histologically confirmed stage II-III gastric adenocarcinoma, as defined by the seventh edition of the American Joint Committee on Cancer (AJCC) tumor-nodes-metastasis (TNM) classification. By multidisciplinary discussion, eligible patients had no marked comorbidities that would preclude the use of adjuvant chemotherapy. After surgery, all patient routinely received 5-fluorouracil-based (5-FU) adjuvant chemotherapy for more than four cycles [19, 20]. In principle, patients were treated until disease progression or unacceptable side effects occurred. Adjuvant chemotherapy was administered by the intravenous route or orally, as appropriate for the specific regimen.

Exclusion criteria were as follows: 1) incomplete clinical and laboratory data; 2) neoadjuvant chemotherapy or radiotherapy; 3) other adjuvant chemotherapy or radiotherapy; 4) preoperative parenteral nutrition before the blood sample was taken. Ultimately, 697 patients were enrolled.

Clinical and laboratory data were retrospectively obtained from an electronic database and the medical records of each patient. The Sun Yat-sen University Cancer Center research ethics committee approved this study that was conducted in accordance with the standards of the Declaration of Helsinki. Informed consent was deemed unnecessary by the Ethical Committee, and all information were anonymous.

### Follow-up strategy

All patients were routinely followed up every 3 months for the first 2 years, every 6 months for the next 3 years, and annually thereafter. Postoperative follow-up procedures included medical checkups, laboratory testing, gastroscope examination, and chest/abdominal computed tomography scan. All patients were monitored either until July 2015 or their death. Median follow-up time was 36 months (range, 3–162 months). Cancer-specific survival (CSS) was calculated from the date of operation until death of GC or last follow-up.

### CONUT score and other markers

Preoperative blood samples were collected and assayed within 2 weeks before surgery. Preoperative CONUT scores were summarized in Table 1 [17]. We set 3 as the optimal cutoff value for CONUT score by receiver operating characteristics (ROC) curves analysis (Additional file 1: Figure S1). BMI, PNI and performance status were calculated and classified based on previous studies [18, 21]. Patients with a combined albumin (g/L) × total lymphocyte count × 10^9^/L ≥ 45 were allocated a PNI score of 0. Patients in whom this total score was < 45 were allocated a score of 1, where a PNI of 1 is indicative of severe nutritional impairment and PNI of 0 is normal [21]. According to the manufacturer’s instructions, the cutoff values for elevated concentrations of serum carcinoembryonic antigen (CEA), carbohydrate antigen (CA) 19–9 and CA 72–4 were 5 ng/mL, 27 U/mL, and 5 U/mL, respectively.

**Table 1:** Assessment of the nutritional status according to the CONUT score

	None	Light	Moderate	Severe
Serum albumin (g/dL)	≥3.50	3.00–3.49	2.50–2.99	< 2.50
Score	0	2	4	6
Total lymphocyte count (/mm^3^)	≥1600	1200–1599	800–1199	< 800
Score	0	1	2	3
Total cholesterol (mg/dL)	> 180	140–179	100–139	< 100
Score	0	1	2	3
CONUT score (total)	0–1	2–4	5–8	9–12
Classification (total score)	≤2 Low CONUT group			
	≥3 High CONUT group			

Abbreviations: *CONUT* controlling nutritional status

### Statistical methods

Our research adhered to the Strengthening the Reporting of Observational Studies in Epidemiology (STROBE) statement (Additional file 2: Table S1). The CSS rate was estimated by the Kaplan–Meier method with the log-rank test. Differences between groups were examined using the Chi-square test for categorical variables. The optimal cutoff value was determined by the maximum of Youden index (sensitivity+ specificity-1) based on ROC curve analyses. The variables in which *p* value was less than 0.05 in the univariate analysis were entered into a final multivariate Cox proportional hazards model to identify independent prognostic factors. A two-sided *P* value < 0.05 was considered to be statistically significant. All of the statistical analyses were performed with SPSS version 19.0 (SPSS, Chicago, IL, USA). All data in our study have been recorded at Sun Yat-sen University Cancer Center for future reference (number RDDA2018000485).

## Results

**Fig. 1:**
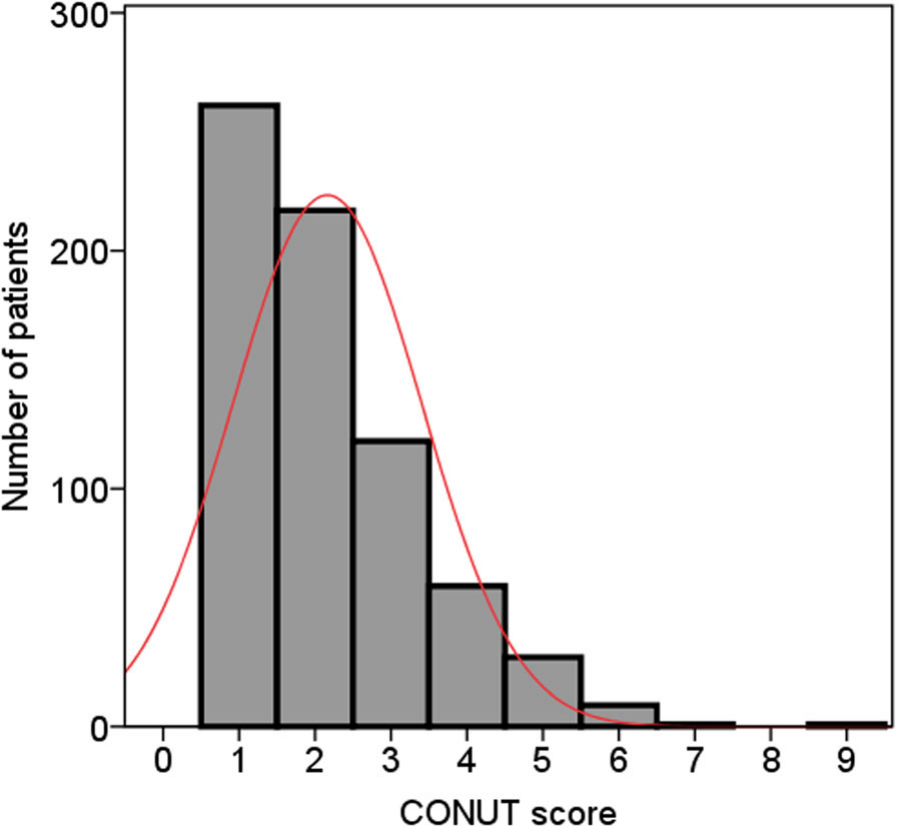
Distribution of the CONUT scores. The histograms of all patients were normally distributed. CONUT = controlling nutritional status

**Fig. 2:**
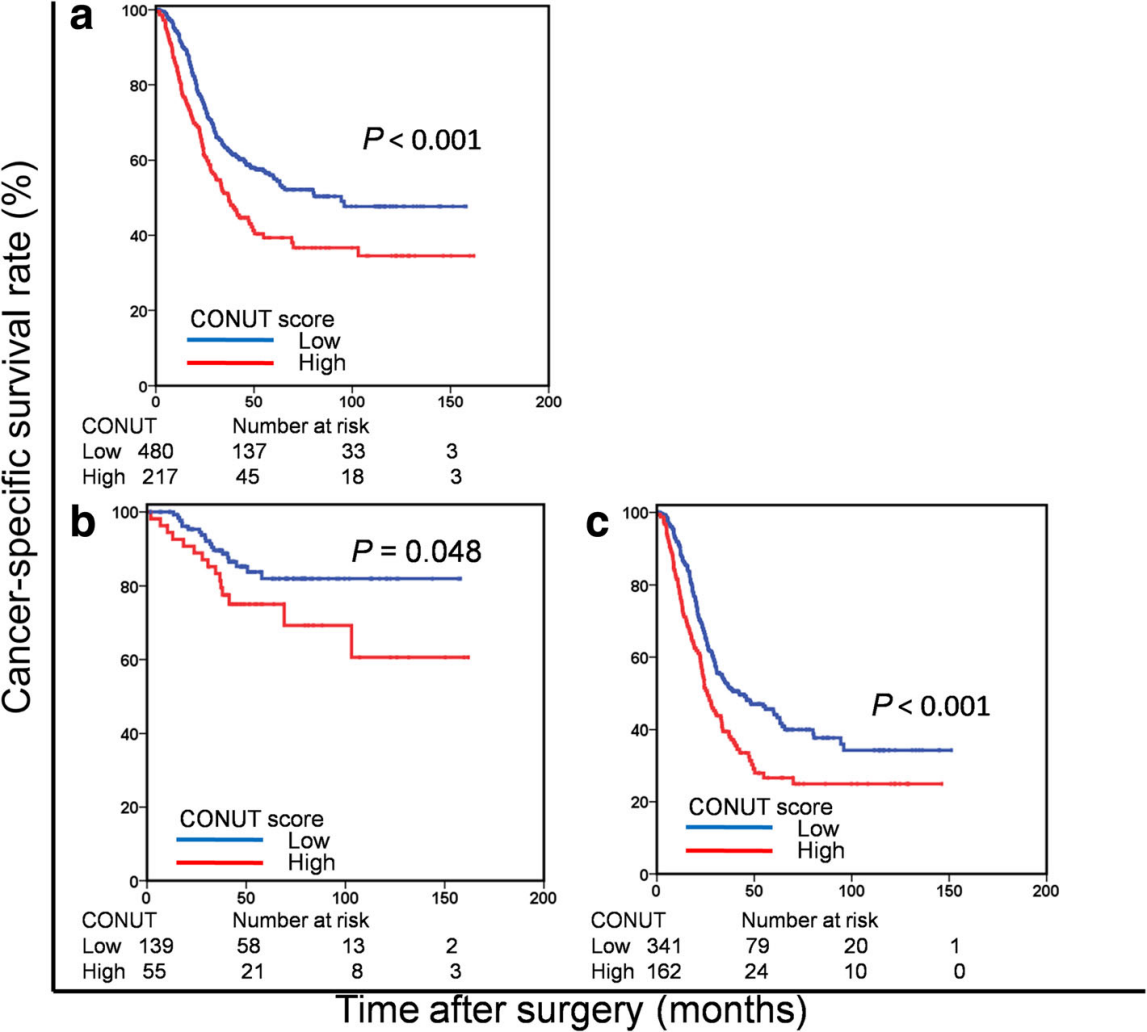
Cancer-specific survival based on the CONUT score in patients with stage II-III (**a**), stage II (**b**), and stage III (**c**) gastric cancer, respectively. CONUT = controlling nutritional status

Of the 697 enrolled patients, 194 (27.8%) were classified as stage II and 503 (72.2%) as stage III. The patient cohort included 457 (65.6%) male patients, with a median age of 57 years (range, 21–86 years) and the mean age was 66.0 years (range 41–89 years). According to the nutritional status in CONUT score, the patients were divided into four groups: none (261 patients, 37.4%), light (396 patients, 56.8%), moderate (39 patients, 5.6%), and severe (1 patients, 0.1%) (Table 1; Fig. 1). Finally, 480 (68.9%) patients were classified into the low CONUT group and 217 (31.1%) patients were classified into the high CONUT group based on a cut-off CONUT value of 3. The Kaplan-Meier curve comparing the CSS of the patients according to the CONUT score is shown in Fig. 2.The high CONUT group had a significantly lower 5-year CSS rate than the low CONUT group (39.3 vs. 55.5%, *P* < 0.001).

The correlation between the CONUT and the clinicopathological factors is shown in Table 2. High CONUT group was significantly associated with larger tumor size (*P* = 0.002), more lymph node metastasis (*P* = 0.010), lower BMI (*P* = 0.009), higher PNI (*P* < 0.001) and the presence of preoperative anemia (*P* < 0.001).

**Table 2:** The clinicopathological characteristics stratified by the CONUT score

	Low CONUT group	High CONUT group	*P* value
	(*n* = 480)	(*n* = 217)	
Age (years)			0.070
< 60	296	118	
≥ 60	184	99	
Sex			0.247
Female	172	68	
Male	308	149	
Tumor size (cm)			0.002
< 5	261	91	
≥ 5	219	126	
Tumor location			0.062
Lower third	183	99	
Upper/Middle third	297	118	
Histological grade			0.053
Well differentiated	56	37	
Poorly differentiated	424	180	
Lauren histotype			0.403
Intestinal	103	59	
Diffuse / Mixed	221	107	
LVI			0.096
Absent	362	106	
Present	27	14	
pT stage			0.134
pT1/2	45	13	
pT3/4	435	204	
pN stage			0.010
pN0/1	190	64	
pN2/3	290	153	
Dissected lymph nodes			0.090
≤ 29	355	147	
> 29	125	70	
TNM stage			0.324
II	139	55	
III	341	162	
Operation type			0.109
Subtotal	332	163	
Total/extended	148	54	
Complications			0.161
No	375	159	
Yes	105	58	
Performance status			0.527
0	119	49	
1/2	361	168	
BMI (Kg/m2)			0.009
< 18.5	294	110	
18.5≤	186	107	
PNI			< 0.001
≥ 45	480	137	
< 45	0	80	
Anemia			< 0.001
No	378	110	
Yes	102	107	
CEA			0.164
Normal	381	162	
Elevated	99	55	
CA19–9			0.633
Normal	375	166	
Elevated	105	51	
CA72–4			0.738
Normal	364	161	
Elevated	116	56	

Abbreviations: *CONUT* controlling nutritional status, *LVI* lymphatic vessel infiltration, *TNM* tumor-node-metastasis staging, *BMI* body mass index, *PNI* prognostic nutritional index, *CEA* carcinoembryonic antigen, *CA* carbohydrate antigen

The results of univariate analyses showed that age, tumor size, tumor location, lymphatic vessel infiltration (LVI), pT stage, pN stage, TNM stage, operation type, PNI, CONUT, CEA, CA19–9, and CA72–4 were associated with CSS (All *P* < 0.05; Table 3). Considering that pT/pN stages were significantly associated with TNM stage, we didn’t include them in the final multivariable analysis. When a multivariate analysis was performed, CONUT score were independent predictors of CSS (HR: 1.553; 95% CI: 1.080–2.232; *P* = 0.017), along with tumor location, LVI, TNM stage and CA19–9.

**Table 3:** Univariate and multivariate analyses of prognostic factors associated with cancer-specific survival

	Univariate analysis	Multivariate analysis
	HR (95% CI) *P*-value	HR (95% CI) *P*-value
Age (years)	0.024	0.421
< 60	1.00	1.00
≥ 60	1.292 (1.035, 1.613)	1.130 (0.839, 1.522)
Sex	0.252	
Female	1.00	
Male	0.875 (0.695, 1.100)	
Tumor size (cm)	< 0.001	0.997
< 5	1.00	1.00
≥ 5	1.505 (1.204, 1.880)	0.999 (0.737, 1.356)
Tumor location	< 0.001	0.027
Lower third	1.00	1.00
Upper/Middle third	1.524 (1.209, 1.922)	1.453 (1.044, 2.022)
Histological grade	0.340	
Well differentiated	1.00	
Poorly differentiated	1.179 (0.840, 1.655)	
Lauren histotype	0.235	
Intestinal	1.00	
Diffuse / Mixed	0.859 (0.669, 1.104)	
LVI	< 0.001	0.041
Absent	1.00	1.00
Present	1.602 (1.299, 1.977)	1.294 (1.011, 1.657)
pT stage	0. 001	
pT1/2	1.00	
pT3/4	2.960 (1.573, 5.571)	
pN stage	< 0.001	
pN0/1	1.00	
pN2/3	3.743 (2.734, 5.124)	
Dissected lymph nodes	0.118	
≤ 29	1.00	
> 29	1.107 (0.974, 1.258)	
TNM stage	< 0.001	< 0.001
II	1.00	1.00
III	4.597 (3.218, 6.567)	4.625 (2.883, 7.421)
Operation type	0.015	0.669
Subtotal	1.00	1.00
Total/extended	1.340 (1.058, 1.697)	1.083 (0.752, 1.558)
Complications	0.755	
No	1.00	
Yes	0.953 (0.701, 1.293)	
Performance status	0.548	
0	1.00	
1/2	0.924 (0.716, 1.194)	
BMI (Kg/m2)	0.159	
< 18.5	1.00	
18.5≤	1.173 (0.940, 1.464)	
PNI	< 0.001	0.085
≥ 45	1.00	1.00
< 45	1.777 (1.313, 2.404)	1.505 (0.945, 2.396)
Anemia	0.231	
No	1.00	
Yes	1.155 (0.912, 1.463)	
CONUT	< 0.001	0.017
≤ 2	1.00	1.00
≥ 3	1.576 (1.255, 1.978)	1.553 (1.080, 2.232)
CEA	0.009	0.269
Normal	1.00	1.00
Elevated	1.407 (1.087, 1.822)	1.222 (0.857, 1.742)
CA19–9	0.002	0.033
Normal	1.00	1.00
Elevated	1.555 (1.183, 2.042)	1.427 (1.029, 1.979)
CA72–4	0.012	0.476
Normal	1.00	1.00
Elevated	1.447 (1.086, 1.927)	1.125 (0.814, 1.555)

Abbreviations: *LVI* lymphatic vessel infiltration, *TNM* tumor-node-metastasis staging, *BMI* body mass index, *PNI* prognostic nutritional index, *CONUT* controlling nutritional status, *CEA* carcinoembryonic antigen, *CA* carbohydrate antigen

**Fig. 3:**
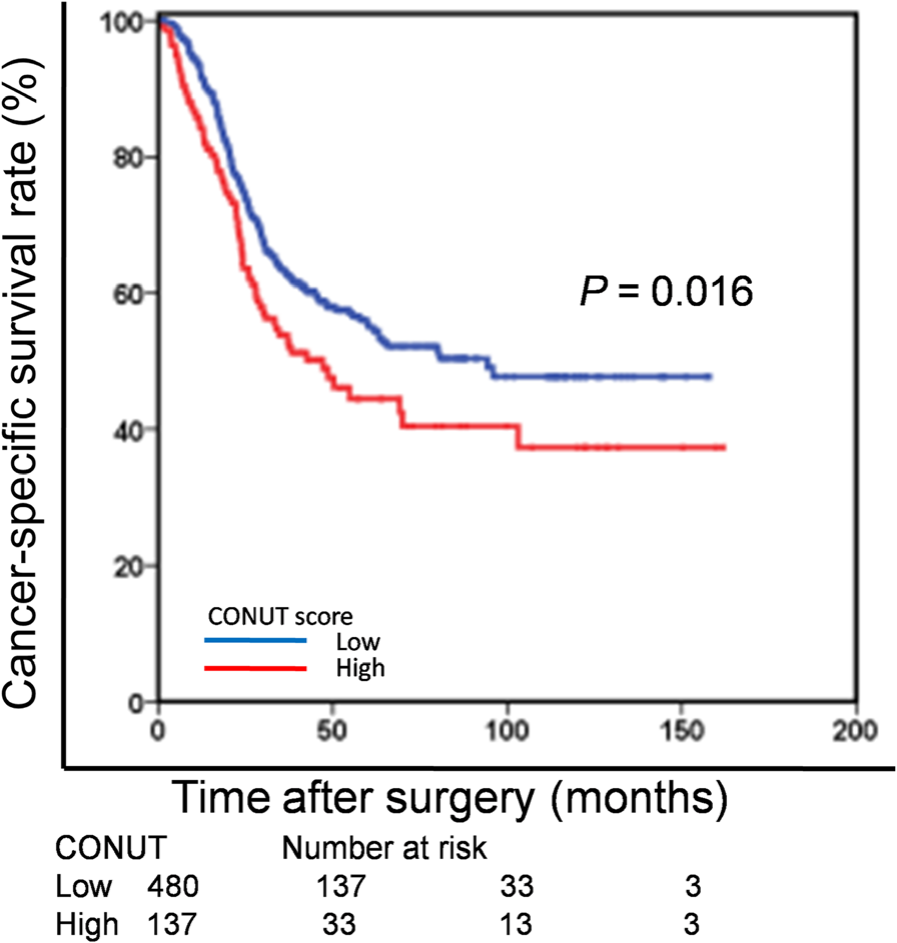
Cancer-specific survival based on the CONUT score in the low PNI group. CONUT = controlling nutritional status; PNI = Prognostic Nutritional Index

When stratified by TNM stage, the prognostic significance of CONUT score was also maintained in patients with stage II (*P* = 0.048) and stage III (*P* < 0.001) GC. Furthermore, we found that 137 patients (22.2%) belonged to the low PNI group and the high CONUT group. Of note, in the low PNI group, CONUT score still effectively stratified CSS (*P* = 0.016; Fig. 3).

## Discussion

Cancer-associated malnutrition is a common but usually unemphasized problem, especially in gastrointestinal malignancies [22]. Increasing evidence has been gathered by clinicians suggesting that malnutrition is closely associated with various clinical consequences, including poor life quality, decreased response to chemotherapy, and the incidence of severe toxicity during adjuvant therapy. Subsequently, severe adverse events often result in decreased oral food intake, treatment schedule modification or interruptions, and greater impairment of life quality, which lead to further malnutrition [23]. In fact, in recent years, it has been well acceptable that malnutrition is associated with poor clinical outcomes [24]. Therefore, clinicians also continue to seek reliable biomarkers for identifying cancer-associated malnutrition and improving the clinical management.

Recently, the presence of immune-nutritional status, as indicated by the CONUT score, has been reported to independently predict prognosis in many malignancies [25]. In present study, we determined the prognostic value of the preoperative CONUT score, along with several common nutritional markers including PNI, BMI, performance status and preoperative anemia, in stage II-III GC patients receiving adjuvant chemotherapy. We found the CONUT score was a independent predictor of outcome in these patients, which appeared to be a superior prognostic marker compared with the other nutritional markers we tested.

Recently, a Japanese study reported, in a series of 416 GC, that CONUT score was retained as an independent prognostic marker in pStage I-II, but not in pStage III GC patients. It should be noted that, in this study, most of patients were early GC and only 14.4% patients were classified as pStage III [18]. As we all know, GC in Japan is often detected at an early stage and has less aggressive clinicopathological features and better prognosis than those from China [26]. Furthermore, under the Japanese social security system, there are fewer problems of cancer-associated malnutrition and unaffordable medical care in Japan. Therefore, our study is needed to further validate the prognostic value of CONUT score in China.

In fact, our conclusions are supported by other studies. Iseki Y et al. reported that the CONUT score was a strong independent predictor of outcomes among colorectal cancer patients and it more accurately predicted prognosis in those patients than the PNI [27]. The PNI, as a promising immune-nutritional index, has previously been reported in many malignancies, including GC. In our study, we also observed that, CONUT score was able to detect more patients who would have a poor survival but not be identified by PNI. As shown in Table 2, we found that 137 patients (22.2%) belonged to the low PNI group and the high CONUT group. Of note, in the low PNI group, CONUT score still effectively stratified CSS. Therefore, in the context of stage II-III GC, the CONUT score might exert more potent prognostic effect than did the PNI. This is partly attributed to the fact that there is greater emphasis placed on the total lymphocyte count in the CONUT score. Furthermore, total cholesterol concentration which is not evaluated in the PNI may play an important role as part of the CONUT score composite measure. Therefore, we speculated that the CONUT score might be a more comprehensive and superior predictor to identify nutritional risk than the PNI in GC. Maehara et al. enrolled 109 patients with lung cancer with obstructive pulmonary disease and found the CONUT score was an independent predictor of disease-free and overall survival [28]. Likewise, Maehara et al. evaluated and reported the prognostic value of CONUT score in 357 patients with hepatocellular carcinoma. They found that CONUT score was independently associated with overall survival, but not recurrence-free survival, in hepatocellular carcinoma patients undergoing curative resection [29].

Based on our study, it is thought that the preoperative CONUT score may be useful in the stratification of risk and tailoring individualize treatments. In clinical practice, patients with high CONUT score should receive more effective adjuvant therapy and shorten the follow-up interval. Furthermore, considering the promising results of targeted nutritional intervention, patients with high CONUT score may benefit from preoperative nutritional intervention [30–32]. However, up to now, the optimum nutritional intervention for improving the cancer-associated malnutrition has yet to be established. With all this in mind, we suggest that preoperative nutritional support based on the CONUT score should be evaluated in prospective randomized controlled studies.

Some limitations associated with our study warrant mention. First, it was a retrospective single-center rather than multicenter study. Thus, there might be potential selection bias for the inclusion of patients. Second, we did not have information on postoperative CONUT score and surgical complications. Future studies are needed to further explore. Third, different nutritional support after surgery was inevitable, and this might have confounded our results.

## Conclusions

The CONUT score is independently associated with CSS in patients undergoing curative surgery followed by adjuvant chemotherapy for stage II-III GC. As a convenient, objective and noninvasive marker, it may be useful for treatment decision-making and improving follow-up performance.

## Additional files

Additional file 1: **Figure S1.** Receiver operating characteristics curve for the CONUT score. CONUT = controlling nutritional status (TIF 2636 kb).

Additional file 2: **Table S1.** STROBE Statement—Checklist of items that should be included in the study (DOC 79 kb).

## Abbreviations

AJCC: American Joint Committee on Cancer
BMI: body mass index
CA: carbohydrate antigen
CEA: carcinoembryonic antigen
CI: confidence interval
CONUT: controlling nutritional status
CSS: cancer-specific survival
CT: computed tomography
GC: gastric cancer
HR: hazard ratio
LVI: lymphatic vessel infiltration
PNI: prognostic nutritional index
ROC: receiver operating characteristic
STROBE: Strengthening the Reporting of Observational Studies in Epidemiology
TNM: tumor–nodes–metastasis
