# Supplementary figures and images for: Preoperative controlling nutritional status (CONUT) score as a predictor of long-term outcome after curative resection followed by adjuvant chemotherapy in stage II-III gastric Cancer
Source: BMC Cancer. 2018 Jun 28;18:699. doi: 10.1186/s12885-018-4616-y (PMC6022496; doi:10.1186/s12885-018-4616-y)

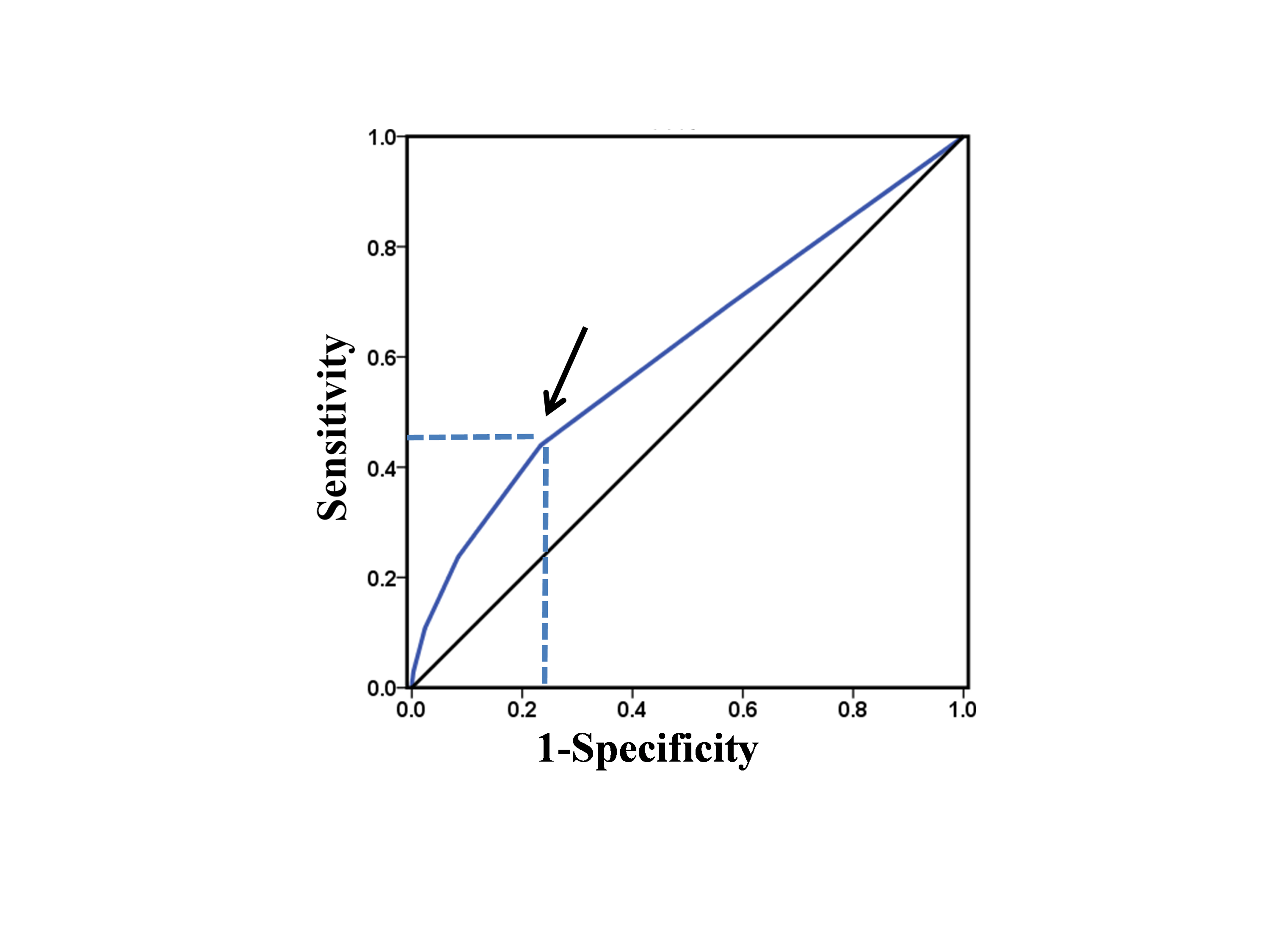

Supplement: Supplementary file 1 — Figure S1. Receiver operating characteristics curve for the CONUT score. CONUT = controlling nutritional status (TIF 2636 kb). [file 12885_2018_4616_MOESM1_ESM.tif]
